# Impact of Endobronchial Ultrasound (EBUS) Training on the Diagnostic Yield of Conventional Transbronchial Needle Aspiration for Lymph Node Stations 4R and 7

**DOI:** 10.1371/journal.pone.0153793

**Published:** 2016-04-15

**Authors:** Inderpaul Singh Sehgal, Sahajal Dhooria, Nalini Gupta, Amanjit Bal, Babu Ram, Ashutosh Nath Aggarwal, Digambar Behera, Ritesh Agarwal

**Affiliations:** 1 Department of Pulmonary Medicine, Postgraduate Institute of Medical Education and Research, Chandigarh, India; 2 Department of Cytology and Gynecological Pathology, Postgraduate Institute of Medical Education and Research, Chandigarh, India; 3 Department of Histopathology, Postgraduate Institute of Medical Education and Research, Chandigarh, India; V.P.Chest Institute, INDIA

## Abstract

**Background:**

There is sparse literature on whether training in endobronchial ultrasound (EBUS)-guided transbronchial needle aspiration (TBNA) improves the diagnostic yield of conventional TBNA (cTBNA).

**Objectives:**

The aim of this study was to evaluate the diagnostic yield of cTBNA before and after the introduction of EBUS.

**Methods:**

This was a retrospective analysis of patients who underwent cTBNA at our center. The study was divided into two periods, before and after the introduction of EBUS at our facility. The diagnostic yield of cTBNA was compared between the study periods. Rapid on-site cytological examination was not available.

**Results:**

A total of 1,050 patients (61.6% men; mean age 45.6 years) underwent cTBNA during the study period (849 before EBUS; 201 after EBUS). Sarcoidosis (n = 527) followed by bronchogenic carcinoma (n = 222) formed the most common indications for performing cTBNA. There was a significant increase in both the success of obtaining a representative sample (from 71% to 85%), and the diagnostic yield (from 33% to 49.5%) of cTBNA, after the introduction of EBUS. The increase in the diagnostic yield of cTBNA after introduction of EBUS remained significant even after adjusting for years of performing cTBNA and the type of anesthesia (topical vs. sedation and topical) on a multivariate analysis.

**Conclusion:**

The diagnostic yield of cTBNA at our facility increased after the introduction of EBUS-TBNA. However, given the retrospective nature of the study, prospective studies are required to confirm our findings.

## Introduction

Conventional transbronchial needle aspiration (cTBNA) is a bronchoscopic procedure that samples accessible hilar and mediastinal lymph nodes with the help of bronchial anatomic landmarks, guided by computed tomography (CT).[1] With the advent of endobronchial ultrasound (EBUS), the use of cTBNA in mediastinal lymph node sampling has declined, especially in the developed countries.[2] EBUS is not only safe but also by its intrinsic advantage of real-time imaging has a higher diagnostic yield and can sample even small sized lymph nodes and lymph node stations that are traditionally considered difficult for cTBNA.[3, 4] On the other hand, cTBNA is also safe, easy to perform and more importantly, it is cost-effective. Thus, it remains an important diagnostic tool in the initial evaluation of mediastinal lymphadenopathy, especially in resource-limited settings.[5–7] The diagnostic yield of cTBNA depends on several factors such as the size and location of the lymph node, number of lymph nodes sampled, and the number of passes.[1, 8] Besides this, the success of cTBNA has been shown to increase after about 30–50 procedures, the so called ‘learning curve’ to attain proficiency in performing TBNA.[9]

Recent evidence suggests that the diagnostic yield (and thus the training) of cTBNA may improve after acquisition of knowledge gained by performing EBUS-TBNA.[10] Herein, we provide results from a large dataset studying the impact of EBUS-TBNA on the diagnostic yield of cTBNA.

## Methods

### Subjects

This was a retrospective analysis of consecutive adult (≥18 years) subjects who underwent cTBNA between 1^st^ January 2006 and 1^st^ April 2015, in the bronchoscopy suite of this Institute. We retrieved the following information from the bronchoscopy database: details of clinical evaluation, tuberculin skin test (TST), findings on chest radiograph and CT of the chest, cytology findings of the aspirate, and the final diagnosis. The study protocol was approved by the Institute Ethics Committee (intramural), and a consent waiver was given as this was use of anonymized retrospective patient data. However, a procedural consent was obtained from all patients. A part of this data has been published previously.[11–14]

### Conventional TBNA procedure

cTBNA is being performed at our facility since 2006 while EBUS-TBNA was started in June 2011.[3, 15] cTBNA procedures were performed by operators under direct supervision of the consultants. The procedure was performed on an outpatient basis till December 2012 under topical anesthesia alone and from January 2013 onwards under topical anesthesia and conscious sedation (intravenous midazolam and pentazocine in doses sufficient to maintain sedation and cough control).[12, 13] Subjects were administered 0.6 mg atropine and 25 mg promethazine intramuscularly followed by nebulized lignocaine (4% solution) immediately before the procedure. Topical 10% lignocaine was sprayed over the oropharynx augmented with 2% lignocaine solution instilled over the vocal cords and the airways.[16] Monitoring of pulse rate, respiratory rate and pulse oximetric saturation was performed throughout the procedure.

A flexible bronchoscope (BF-1T20, BF-TE2, BF-1T150 or BF-IT 180, Olympus, Japan; FB-19TV, Pentax, Japan) was used to perform cTBNA, as described previously.[13] Smears were prepared on glass slides and sent for cytopathological examination and cultures. Rapid on-site cytologic evaluation (ROSE) was not available.

### Interpretation of cTBNA samples

An experienced cytopathologist examined all the smears for adequacy of the samples and the definite diagnosis. TBNA samples were categorized as: (a) diagnostic: if cTBNA enabled a final diagnosis of tuberculosis, sarcoidosis, malignancy, lymphoma and other diagnosis; (b) representative: either by a diagnostic sample or by a preponderance of benign lymphocytes.

Cytologic samples containing malignant cells were considered diagnostic of malignancy. A final diagnosis of sarcoidosis was made on the presence of all the following criteria: (a) consistent clinical and radiological presentation; (b) demonstration of non-necrotizing granulomas on c-TBNA along with negative acid-fast bacilli and fungal stains; and no growth of mycobacteria on MGIT; and, (c) clinical and radiological response after treatment with glucocorticoids. A diagnosis of tuberculosis was based on the demonstration of all the following: (a) necrotizing granulomatous inflammation or presence of acid-fast bacilli (AFB) on microscopy or a positive culture for *Mycobacterium tuberculosis*; and, (b) clinicoradiological response to anti-tuberculosis treatment.

The study was divided into two periods: period I (before the introduction of EBUS) and period II (after the introduction of EBUS). The first 100 subjects who underwent c-TBNA after initiation of the EBUS facility were included in period I as these procedures were conducted in a period considered to be the learning curve of EBUS training. The yield of cTBNA technique was analyzed during the two periods with regards to representative sampling of lymph node and the diagnostic yield. We however, also reanalyzed the data by including the initial 100 cases done after introduction of EBUS period (‘learning curve’ cases) in period II.

### Statistical analysis

Statistical analysis was performed using the commercial statistical package StatsDirect (Version 2.8.0, England, StatsDirect Ltd, www.statsdirect.com). Data were expressed as mean ± standard deviation (SD), or number with percentage. Differences between continuous variables in the two groups were compared using Mann-Whitney U test while differences between categorical data were compared using the chi-square test or Fisher’s exact test. A multivariate logistic regression analysis was performed to study the effects of EBUS training, cTBNA experience and the type of anaesthesia on the diagnostic yield of cTBNA. A p value <0.05 was considered statistically significant.

## Results

A total of 12,044 bronchoscopic procedures were carried out during the study period. Conventional TBNA was performed in 1,050 (8.7%) subjects while EBUS was done in 14.7% (737/5,005) of the subjects. There were 647 (61.6%) men with a mean (SD) age of 45.6 (11) years (Table 1). Of the 1,050 subjects, sarcoidosis (n = 527) followed by bronchogenic carcinoma (n = 222) formed the most common clinical indications for performing cTBNA. Tuberculosis (n = 208) and miscellaneous conditions (n = 85) that included non-resolving pneumonia, fungal pneumonia, and diffuse parenchymal lung disease other than sarcoidosis were the other indications for performing cTBNA. The cTBNA was predominantly performed on lymph node stations 4R and 7 from 2006 to 2011 and almost exclusively in these stations thereafter. The lymph node size data was available for 250 patients (426 lymph nodes). The median (interquartile range) size of lymph node station 4R and 7 was 20 (15.2–28.5) mm and 20 (15–25) mm, respectively. A median of three passes were obtained from each lymph node station. The median (interquartile range) time needed for performing a cTBNA procedure was 15 (13–20) minutes. A final diagnosis of sarcoidosis and tuberculosis was made in 234 and 66 patients, respectively on the basis of results of pathological examination and microbiology while bronchogenic carcinoma was diagnosed in 127 patients.

**Table 1:** Clinical and lymph node characteristics of the patients (n = 1050).

Parameter	Period I (n = 849)	Period II (n = 201)	Total	P value
Age, in years	46 (35–56)	43 (33–54)	45 (35–55)	0.013
Male gender, n (%)	532 (62.7)	115 (57.2)	647 (61.6)	0.153
Clinical diagnosis, n (%)				<0.0001
Tuberculosis	161 (19)	47 (23.4)	208 (19.8)	
Sarcoidosis	404 (47.6)	123 (61.2)	527 (50.2)	
Bronchogenic carcinoma	194 (22.9)	28 (13.9)	222 (21.1)	
Lymphoma	5 (0.6)	2 (1)	7 (0.7)	
Others*	84 (9.9)	1 (0.5)	85 (8.1)	
Lymph node characteristics on CECT thorax (n = 426)				
Size of station 4R, in mm	19.6 (12–36.4)	20.9 (17–28)	20 (15.2–28.5)	0.446
Size of station 7, in mm	20 (15–23.4)	20 (15.3–25)	20 (15–25)	0.582
No. of passes station 4R	3 (2–4)	3 (2–3)	3 (2–3)	0.018
No. of passes station 7	3 (1–3)	3 (2–3)	3 (2–3)	0.080
Time for procedure, in minutes	15 (15–20)	15 (13–20)	15 (13–20)	0.372

All values are specified as median (interquartile range), unless specified

*Others included non-resolving pneumonia, fungal pneumonia, interstitial lung disease, cryptogenic organizing pneumonia and pleural effusion

During period I, 849 patients underwent cTBNA while in period II, 201 patients underwent cTBNA. Presumed sarcoidosis as an indication for performing cTBNA increased from 47.6% to 61.2% from period I to period II. Diagnosis and staging of bronchogenic carcinoma and other conditions as indications showed a decreasing trend from 22.9% to 13.9% and 9.9% to 0.9%, respectively as an indication for performing cTBNA. The size of the lymph nodes (both station 4R and 7) and the time needed to perform the procedure were similar between the study periods. However, the median number of passes at station 4R was significantly higher in period I in comparison to period II.

**Fig 1:**
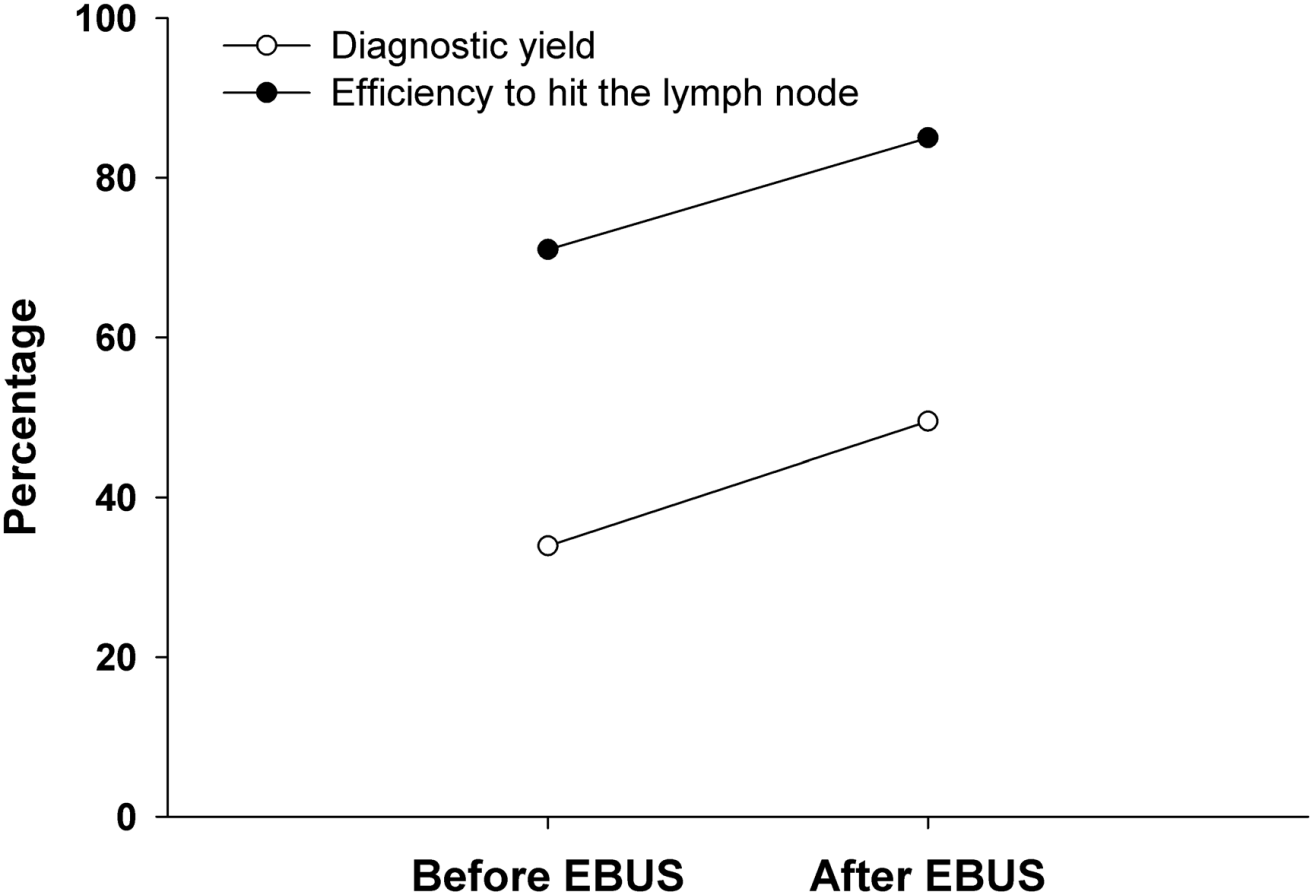
Yield of conventional transbronchial needle aspiration (cTBNA) before and after introduction of endobronchial ultrasound. There was significant increase in the diagnostic yield of cTBNA after introduction of EBUS.

**Fig 2:**
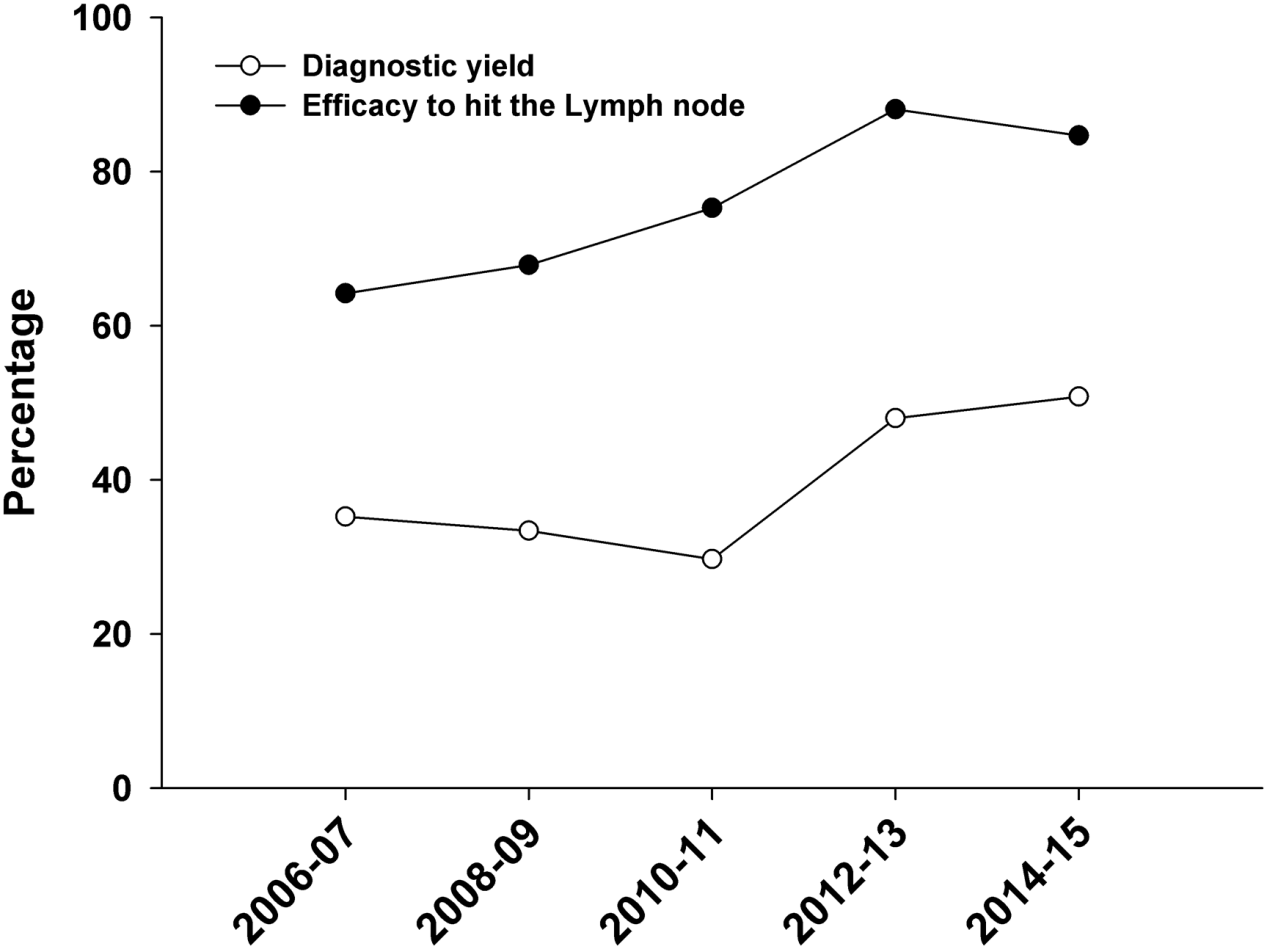
Learning curve of transbronchial needle aspiration (TBNA) over a 10-year period. The proportion of representative lymph node sampling as well as diagnostic yield has increased over time.

Of the total number of bronchoscopic procedures, the proportion of cTBNA performed reduced from 12.1% (849/7039) in period I to 4% (201/5005) in period II. The information on the yield of cTBNA yield was available in 1,048 patients (period I, 848; period II, 200). In two patients, the details of cytological examination were not available and hence they were excluded from the analysis for diagnostic yield. There was a significant increase in the diagnostic yield of cTBNA after the introduction of EBUS (p<0.0001), increasing from 33% to 49.5% (Fig 1). There was also a significant increase in the success of obtaining a representative sample after the introduction of EBUS (p<0.0001, Fig 1). The results did not significantly differ even when the initial 100 cases after introduction of EBUS were included in period II for the analysis of diagnostic yield (Table 2). In the year wise analysis, there was a consistent increase in the diagnostic yield of cTBNA (from 35.2% in 2006–07 to 50.8% in 2014–15), while the proportion of representative samples showed an increasing trend from 64.2% in 2006–07 to 85% in 2014–15 (Fig 2). On a multivariate logistic regression analysis, the diagnostic yield of cTBNA significantly increased in the “after EBUS” period compared to “before EBUS” period after adjusting for the years of performing cTBNA and the type of anaesthesia used (Table 3). The cTBNA procedure was associated with minor complications in five patients (bleeding, n = 4; transient hypoxemia, n = 1).

**Table 2:** Diagnostic yield before and after introduction of EBUS. In this analysis, the initial 100 cTBNA cases after introduction of EBUS are included in the after EBUS period and is further stratified based on the clinical diagnosis.

	Period I (n = 749)	Period II (n = 301)	P value
Overall yield	236 (31.6%)	143 (47.5%)	<0.0001
Clinical diagnosis			
Tuberculosis (n = 208)	31 (24.2%)	40 (50%)	<0.0001
Sarcoidosis (n = 527)	97 (28%)	82 (45.8%)	<0.0001
Malignancy (n = 222)	82 (44.8%)	20 (52.6%)	0.38

**Table 3:** Multivariate analysis of diagnostic yield of conventional transbronchial needle aspiration (cTBNA).

	Adjusted odds ratio (95% confidence intervals)	P value
cTBNA before and after EBUS	1.95 (1.08–3.50)	0.026
Cumulative yield over the years	1.02 (0.94–1.11)	0.555
Conscious sedation vs. topical anesthesia	0.89 (0.48–1.66)	0.891

EBUS: endobronchial ultrasonography

## Discussion

The results of this study suggest that the diagnostic yield of cTBNA at our facility increased from 33% to 49.5% after the introduction of EBUS. The increase in diagnostic yield remained significant even after adjusting for the years of performing cTBNA and the type of anaesthesia (topical anaesthesia vs. conscious sedation and topical anaesthesia) during bronchoscopy. The cTBNA procedure as a proportion of the total bronchoscopic procedures declined from 12% to 4%, after the introduction of EBUS-TBNA. This was also seen in another study where the number of cTBNA procedures declined by almost half from 11.75 cTBNA per month in pre-EBUS era to 6.03 cTBNA per month in the post-EBUS era.[2] The proportion of patients with lung cancer and other indications decreased; however, the proportion of patients with sarcoidosis undergoing cTBNA increased. This is due to the fact that conventional bronchoscopic techniques have a high diagnostic yield in patients with sarcoidosis.[12, 17]

Although cTBNA is an easy and safe procedure to sample the mediastinal lymph nodes, it is underutilized due to the wide variations in the reported success rate and the unfounded fear of causing trauma to major blood vessels.[1, 5] The cTBNA is not a completely ‘blind’ procedure as it is guided by mediastinal lymph nodal location on CT chest; however, it requires a comprehensive understanding of the mediastinal anatomy and development of skill set.[1, 9] First introduced in 2003, EBUS provides real time images and enables the performance of needle aspiration under direct vision.[18] It is probable that training in EBUS with its real time imaging may enhance the understanding of mediastinal anatomy among the operators and thus indirectly helps in improving the skills of performing cTBNA. In a study comprising of 214 patients with non-small cell lung carcinoma, there was significant increase in the diagnostic yield of cTBNA after EBUS training.[10] The overall sensitivity of cTBNA increased from 63% before EBUS to 86% in the after EBUS group.[10] This was also observed in our study in which there was an increase in the representative lymph node sample and the overall diagnostic yield. Our study however, included an unselected group of patients comprising of both malignant and benign respiratory disorders. In contrast, the diagnostic yield of cTBNA did not alter in the pre- and post-EBUS era in another study.[2] However, previous studies have not performed a multivariate analysis wherein other factors such as cumulative experience and the type of anesthesia have been adjusted.

What are the clinical implications of this study? The results of this study suggest that cTBNA remains a useful technique in the diagnosis of mediastinal adenopathy with almost a 50% yield despite multiple operators with varying experience performing the procedure. This is especially important for the developing world, where EBUS-TBNA is not easily available due to constraints of cost while cTBNA can be readily introduced with no added costs. The results of the study do not imply that operators without EBUS misunderstand the mediastinal anatomy and are not correctly aspirating a lymph node station. Rather, the fellows undergoing training perform cTBNA procedures better, due to superior understanding of the mediastinal anatomy after watching and training on EBUS. Thus, if academic institutes acquire EBUS and train their fellows in both the TBNA techniques, it is likely that the yield of cTBNA would increase after performing EBUS-TBNA.

Finally, the study has a few limitations. This is a retrospective time-series analysis, a study design fraught with confounders and bias. The increase in the diagnostic yield could also be due to several factors other than the introduction of EBUS. We implemented a strict cTBNA procedure protocol that was not in place prior to EBUS. Also, we meticulously observed and recorded the data of lymph node location and size for all patients undergoing TBNA (both conventional and EBUS guided), after the introduction of EBUS.[19–21] The increased diagnostic yield may also reflect a selection bias as only patients with significantly enlarged lymph nodes (short axis lymph node size of at least one centimeter on CT scan) at stations 4R and 7 underwent cTBNA, after the introduction of EBUS while those with smaller nodes underwent EBUS-TBNA. The learning curve of the cytopathologist dealing with cTBNA and EBUS-TBNA might have also significantly improved between 2006 and 2015 and could have contributed to the results. It is also possible that inter-individual differences in the skill of different bronchoscopists could have led to differences in the study results. However, the procedure of bronchoscopy was performed by fellows and consultants throughout the study period. Although with years, the skill of the operator is likely to improve but the dynamics of the training program at our centre ensured symmetry throughout the study period. Each year new fellows are inducted and perform bronchoscopy only after training adequately on the simulator, under direct supervision of the consultant. Hence, skills of different operators are very unlikely to affect the differences in the results of the current study. Finally, all these limitations are partially offset by the high procedural volume at our center.

In conclusion, introduction of EBUS-TBNA procedure at a bronchoscopy facility enhances the diagnostic yield of c-TBNA in patients with mediastinal lymphadenopathy. However, due to the retrospective nature of our study, prospective studies are required to confirm our study findings.
